# Nr4A1 mRNA regulation of FSH-induced meiotic resumption in bovine cumulus-oocyte complexes cultured in vitro

**DOI:** 10.5935/1518-0557.20140010

**Published:** 2014

**Authors:** Jessica E. Hicks, Robert M. Petters, Jeffrey R. Sommer, Charlotte E. Farin

**Affiliations:** 1 Department of Animal Science, College of Agriculture and Life Sciences, North Carolina State University - EUA

**Keywords:** AMH, FSH, antral follicles, ovarian reserve, ongoing pregnancy

## Abstract

**Objective:**

1) further define the time course required for transcription initiation in bovine cumulus-oocyte-complexes (COC); 2) determine the pattern of expression for Nr4A1 and Egr1 mRNAs in bovine COC; and, 3) reduce Nr4A1 mRNA expression using small interfering RNAs (siRNA) to determine the effect on breakdown of the germinal vesicle (GVBD)

**Methods:**

A series of experiments were performed to define the time required for transcription initiation during FSH-induced maturation in bovine COCs, determine the pattern of expression for candidate mRNAs during GVBD, and use RNAi to determine their potential role in GVBD by examining whether candidate-specific siRNA can reduce mRNA expression in bovine COCs and affect the occurrence of GVBD.

**Results:**

Transcription required for GVBD in bovine COC occurred as early as 30 min after culture initiation. Expression of Nr4A1 mRNA increased (*P* <0.05) at 30 min after culture initiation, consistent with the time of transcription initiation required for GVBD. Expression of Egr1 mRNA did not differ during culture. Expression of Nr4A1 mRNA was decreased (*P* <0.05) in COC cultured with 50nM siNr4A1 or with 120 µM of the transcriptional inhibitor DRB compared to controls. The proportion of COC undergoing GVBD at 9 hr of culture in FSH and non-specific siRNA (siNS) treatment groups did not differ. However, fewer (*P* <0.05) COC underwent GVBD at 9 hr of culture when in the presence of DRB or 50nM siNr4A1 compared to controls.

**Conclusion:**

these data support a role for Nr4A1 in regulating FSH-mediated and transcription-dependent GVBD in bovine COC cultured in vitro.

## INTRODUCTION

In bovine oocytes, the resumption of meiosis is characterized by the breakdown of the germinal vesicle (GVBD). In vivo, due to an increase in gonadotropin levels, the dictyate oocyte undergoes GVBD, proceeds through meiosis, extrudes the first polar body and is arrested again at the MII stage until fertilization (Jamnongjit & Hammes, 2005; Ferreira *et al.*, 2009). In vitro, oocytes resume meiosis spontaneously when they are removed from the inhibitory follicular environment and cultured in the absence of gonadotropins (Edwards, 1965; Farin & Yang, 1994). In addition to spontaneous maturation, which occurs in the absence of gonadotropins, even in the presence of gonadotropins such as FSH, oocytes can undergo maturation in vitro. However, the time course for spontaneous versus FSH-induced in vitro maturation differ (Farin & Yang, 1994; Rodriguez & Farin, 2004; Farin *et al.*, 2007).

FSH-induced in vitro oocyte maturation is dependent upon an initial transcriptional event that is mediated through the cumulus cells that surround the oocyte (Rodriguez & Farin, 2004). This event is vital for the resumption of meiosis and subsequent GVBD and is believed to occur within the first 1 to 1.5 hrs of culture for bovine cumulus-oocyte-complexes (COC; Osborn & Moor, 1983; Kastrop *et al.*, 1991; Farin & Yang, 1994; Sirard *et al.*, 1998). Serial analysis of gene expression (SAGE) was used to compare mRNA expression profiles from murine COC that underwent FSH-induced oocyte maturation in vitro to those from murine COC that were prevented from undergoing in vitro maturation by culture with the transcriptional inhibitor 5,6-dichloro-1-β-D-ribofuranosyl-benzamidazole (DRB; Rodriguez *et al.*, 2006). Comparisons of SAGE libraries generated from control and inhibitor-treated COC allowed the identification of candidate mRNAs whose expression was temporally associated with the initiation of GVBD in murine COC (Rodriguez *et al.*, 2006). Two of the major candidate mRNAs identified in that experiment were Nuclear Receptor 4 Group A Member 1 (Nr4A1) and Early Growth Response 1 (Egr1). Each of these mRNAs demonstrated a significant decrease in expression in murine COC that were cultured with FSH and the transcriptional inhibitor compared to culture in the presence of FSH alone (Rodriguez *et al.*, 2006). These observations suggest that these two mRNAs may play a role in the transcriptional regulation of FSH-mediated GVBD.

RNA interference (RNAi) allows for the identification of specific mRNA function without requiring preparation of knockout animals (McManus & Sharp, 2002; Sledz *et al.*, 2003). For RNAi, small portions of double-stranded (ds) RNA are used to block the expression of a homologous target mRNA, inducing selective mRNA degradation (Fire *et al.*, 1998). Twenty-one to 23 nucleotide dsRNAs, known as small interfering RNA (siRNA), influence RNAi specificity and act as an intermediate for mRNA silencing (McManus & Sharp, 2002). siRNA treatment has been used to reduce mRNA levels for cyclin B1 (Paradis *et al.*, 2005), mitogen-activating protein kinase (Svoboda *et al.*, 2000; Yu *et al.*, 2007) and c-mos (Svoboda *et al.*, 2000) in cultured oocytes.

The overall objective of this study was to assess the function of the candidate mRNAs, Nr4A1 and Egr1, during FSH-mediated bovine oocyte maturation in vitro. The specific objectives of this study were to: 1) further define the time required for transcription initiation during FSH-induced maturation in bovine COCs; 2) determine the pattern of expression for these two candidate mRNAs during GVBD; and, 3) use RNAi to determine their potential role in GVBD by examining whether candidate-specific siRNA can reduce mRNA expression in bovine COCs and affect the occurrence of GVBD.

## MATERIAL AND METHODS

Tissue culture medium (TCM-199 with Earle’s salts) was obtained from Gibco^®^ (Carlsbad, CA). Follicle stimulating hormone from porcine pituitary glands (50 mg/vial Armour FSH standard) and 5,6-dichloro-1-β-D-ribofuranosyl-benzimidazole (DRB) were purchased from Sigma-Aldrich, Inc. (St. Louis, MO). All other reagents for culture medium were of tissue culture grade and were obtained from Sigma-Aldrich, Inc. (St. Louis, MO). TRI Reagent^®^ for RNA extractions was purchased from Molecular Research Center Inc. (Cincinnati, OH). DNase I and Taq DNA polymerase were obtained from Roche Applied Science (Indianapolis, IN). SuperScript^®^ III RT, dNTPs, TOPO® Cloning Kit, Lipofectamine 2000™, and Opti-MEM^®^ I Medium were purchased from Invitrogen (Carlsbad, CA). QIAquick PCR Purification Kit, Qiaex II Kit, and QIAprep Spin Miniprep Kits were obtained from Qiagen, Inc. (Valencia, CA). SMARTpool^®^ custom siRNA pools were synthesized and purchased from Dharmacon Research, Inc. (Lafayette, CO).

### Oocyte Recovery and Culture Conditions

Ovaries were harvested from a local abattoir and transported to the laboratory in roller bottles containing 0.9% saline. Ovaries were then rinsed 3 times with saline and follicles between 2 and 10 mm in diameter were aspirated using an 18 ga needle and syringe. Cumulus-oocyte-complexes with at least 3 complete cumulus cell layers were collected, washed 3 times in modified Tyrode’s medium (TL-Hepes; Parrish *et al.*, 1986) and placed into appropriate treatment groups containing 0.8ml TCM-199 supplemented with 10% heat-inactivated estrous cow serum, 1µg/ml estradiol, 200nM pyruvate, 5µg/ml FSH, and 50µg/ ml gentamicin (if applicable). All cultures were maintained at 38.5°C in an atmosphere of 5% CO2 in air with 100% relative humidity. When applicable, treatment groups contained 120µM DRB in 0.2% dimethyl sulfoxide (DMSO). Medium containing DRB was replaced at 4hr intervals throughout culture. At appropriate time points, COC were either snap frozen in liquid nitrogen and stored at-80ºC prior to RNA extraction or placed on slides for assessment of their stage of meiotic maturation.

### Assessment of Meiotic Stage

At appointed times, COC were removed from culture, placed in tubes with 0.4ml TL-Hepes and vortexed for 4 min 30 sec to remove cumulus cells. Oocytes were then washed with phosphate-buffered saline (PBS), placed on acid-washed slides and fixed in an ethanol-acetic acid (3:1 v/v) solution for 9 to 24hr. Oocytes were stained with a 1% orcein in 25% acetic acid stain, de-stained with ethanol-acetic acid, and evaluated for meiotic stage using differential interference contrast microscopy at 200X magnification (Farin & Yang, 1994).

### wcRNA Extraction, Reverse Transcription and cDNA Synthesis

TRI Reagent^®^ was added to COC frozen at-80ºC at a ratio of 800µl TRI Reagent^®^ to 150 COCs. Whole cell RNA (wcRNA) extraction was performed according to the manufacturer’s instructions and the resulting wcRNA was resuspended in diethyl pyrocarbonate-treated (DEPC) water.Whole cell RNA concentration was determined by absorbance at 260 nm and quality was assessed by examination of 18S and 28S bands following agarose gel electrophoresis.

Whole cell RNA (2µg) was thawed on ice and treated with 60U DNase I in a 5.5µl reaction for 20 min at 37ºC. This DNA-free wcRNA was then used for primer annealing in a reaction consisting of 250ng random primers, 1µl 10mM dNTP mix, and sterile DEPC water to a volume of 14µl and was incubated at 65ºC for 5 min. The primer-annealed RNA was placed on ice for 1 min and then reverse transcribed at 50ºC for 1 hour in a 20µl reaction consisting of 4µl 5X First Strand Buffer, 1µl 0.1M DTT and 200U of SuperScript^®^ III RT. Reactions were inactivated by heating to 70ºC for 15 min. All cDNA samples were then cleaned using the QIAquick PCR Purification kit as per manufacturer’s instructions and stored at 4ºC in 50µl Qiagen PE buffer (Valencia, CA) until further use.

### Semi-quantitative PCR Assay

Primers used for semi-quantitative PCR (sqPCR) analyses were designed using the Gene Amplify 1.2 (Madison, WI) and Oligo 4.0.2 primer analysis software (Plymouth, MA) using the predicted bovine and murine sequences for either Nr4A1 or Egr1. Primers sets for the Nr4A1 and Egr1 mRNAs were designed to cross introns. Primers for glyceraldehyde-3-phosphate dehydrogenase (Gapdh) mRNA were synthesized based on previously published sequences (Leutenegger *et al.*, 2000). Expression of Gapdh mRNA was used as a housekeeping control. Specific annealing temperatures, cycle numbers for linear amplification and product sizes are presented in Table 1.

**Table 1:** Primer sequences used for semi-quantitative PCR analyses.

mRNA ID	Primer Sequence (5’ to 3’)	Product Size (bp)	Annealing Temp. (°C)	Cycle No.*
Nr4A1	F: GGCTTTGCTGAACTGTCT R: GTCGGTCTGTGATGAGGA	271	62	25
Egr1	F: TGGCTCCTTTCCTCATTC R: TTTGGCTGGGGTAACTCG	317	60	33
Gapdh**	F:GGCGTGAACCACGAGAAGTATAA R: CCCTCCACGATGCCAAAGT	120	60	25

* Cycle number identified to be within the linear range of amplification

** Primers from Leutenegger *et al*., 2000

For sqPCR analysis, PCR reactions contained 2µl 10X buffer (10mM Tris-HCL, 1.5mM MgCl2, 50mM KCl), 2µl (200ng) appropriate forward primer, 2µl (200ng) corresponding reverse primer, 16µM dNTPs, 0.25µl Taq polymerase, 2µl (100ng) cDNA and DEPC water to a final reaction volume of 20µl. Reactions using Nr4A1, Gapdh, and Egr1 primers also included 4µl Q solution (Qiagen, Inc.) with a corresponding decrease in DEPC water to maintain a 20ul reaction volume. All reactions were placed in a 96-well PCR plate, sealed using ThermalSeal™ (Excel Scientific, Wrightwood, CA), and placed into a PTC100 thermal cycler for 2 min at 94ºC. The PCR programs for Nr4A1 and Gapdh used 10 sec steps for denaturation at 92ºC and primer extension at 72ºC. For Egr1, 15 sec steps were used. PCR products were electrophoresed on 1.5% ethidium bromide-stained agarose gels and band intensities were assessed using an AlphaImager^®^ Analysis System (Alpha Innotech, San Leandro, CA). Relative signal intensity was calculated as the ratio of the band intensity of the target product to that of Gapdh mRNA. Data for determination of Nr4A1 siRNA dose and the effects of Nr4A1 siRNA treatment on oocyte maturation were expressed as a percentage of the FSH control treatment.

### Recovery of PCR Products, Subcloning and Sequence Verification

Bands representing PCR amplicons for Nr4A1, Egr1 and Gapdh were extracted from the agarose gel using the Qiaex II kit (Qiagen, Inc.) as per manufacturer’s instructions. Four µl of purified PCR product was subcloned into a TOPO^®^ vector according to manufacturer’s instructions (TOPO^®^ Cloning Kit, Invitrogen). Briefly, 2µl of ligation reaction were added to 50µl electro-competent E. coli cells, mixed gently and electroporated at 1500V 1 time for 3 to 4 milliseconds. Electroporated cells were shaken at 37ºC for 1 hr in 250µl super optimal broth (SOC) medium at 200rpm. Ten, 30, and 50µl aliquots were then placed on Luria broth-ampicillin (LB-Amp) plates and incubated at 37ºC for approximately 15hr. Individual colonies were transferred into 5ml LB broth with 10µl of 50mg/ml ampicillin stock and incubated for approximately 15 hr at 37ºC. Plasmids containing the inserts of interest were purified using the QIAprep Spin Miniprep Kit (Qiagen) and were used as a sequencing template to verify that the PCR amplicons generated represented their intended target sequences. All PCR amplicons were verified to be representative of their intended sequences.

### siRNA Treatment Conditions

Custom SMARTpool^®^ siRNAs (GE Dharmacon, Lafayette CO, USA) were generated from the predicted bovine sequences for Nr4A1 and Egr1. A non-specific siRNA control (siNS) was also generated from unrelated (non-mammalian) sequences provided by the SMARTpool^®^ manufacturer (GE Dharmacon). Small interfering RNA stocks were diluted in 1X working buffer to a 40nM/µl volume, aliquoted in 25µl doses, and frozen at-80ºC until further use. For all culture experiments containing siRNAs, Lipofectamine 2000™ in Opti-MEM^®^ I Reduced Serum Medium was used as a transfection reagent as per manufacturer’s instructions. All cultures containing siRNAs were maintained in the absence of antibiotics. Cumulus-oocyte-complexes were placed into tubes containing the appropriate dose of siRNA and were centrifuged twice at 55g for 3min to improve siRNA transfection. Transfected COC were then placed into appropriate treatment wells and cultured for the appropriate duration. Following culture, COC were either snap-frozen in liquid nitrogen and stored at-80ºC until extracted for wcRNA or mounted on slides for assessment of meiotic stage.

### Overall Experimental Design

A series of experiments were performed to define the time required for transcription initiation during FSH-induced maturation in bovine COCs, determine the pattern of expression for candidate mRNAs during GVBD, and use RNAi to determine their potential role in GVBD by examining whether candidate-specific siRNA can reduce mRNA expression in bovine COCs and affect the occurrence of GVBD. Specific experimental designs for each objective are included within each subsection of the results section. Details concerning the numbers of COC per treatment and number of experimental replications are included in each figure legend. Within each experiment, a positive control treatment, consisting of COC cultured from time zero in the presence of FSH, and a negative control treatment, consisting of COC cultured from time zero in the presence of both FSH and the transcriptional inhibitor, DRB was included. It was expected that >95% of COC would undergo GVBD in the presence of FSH whereas <10% of COC would undergo GVBD in the presence of both FSH and DRB. Additional treatment groups were included depending on the objectives of the specific experiment being described. When assessed, the incidence of GVBD was determined at 9hr of culture.

### Statistics

All data were analyzed by one-way ANOVA and means were separated using Duncan’s tests. Data used to determine effective Nr4A1 siRNA dose and the effects of Nr4A1 siRNA treatment on oocyte maturation were arcsin-transformed. All data are presented as least squares means ± SEM. Results were considered significant at *P* < 0.05.

## RESULTS

### Time Course for Transcription Initiation during Gonadotropin-Induced Maturation

**Figure 1:**
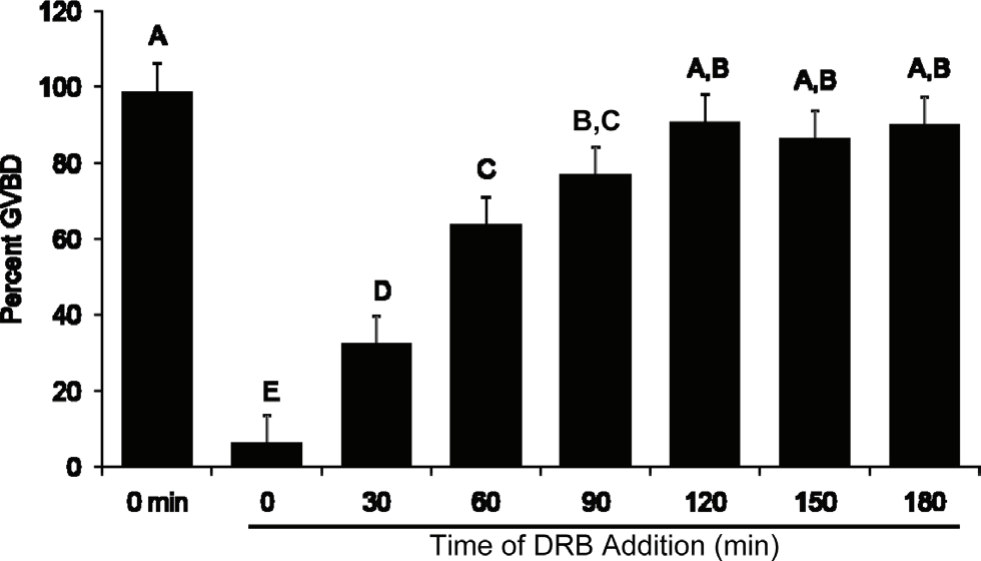
Time course for transcription initiation required for FSH-induced oocyte maturation. Data represent the percent of COC that underwent GVBD at 9h of culture (lsmeans ± SEM; n = 5 replicate experiments with n = 27 ± 2 COC/treatment per replicate);^A,B,C,D,E^ *P* <0.05).

To identify the time during which transcription is required for GVBD, the transcriptional inhibitor, DRB, was added at initiation of culture or at 30, 60, 90, 120, 150 and 180 min after the start of culture. In all of these treatment groups, FSH was present throughout the culture period. COC were harvested after 9 hr of culture to assess the incidence of GVBD. Consistent with previous studies, when COC were cultured entirely in the presence of DRB fewer oocytes underwent GVBD (6.4 ± 7.2%) compared to the 0 min (untreated) controls that contained FSH alone (99.0 ± 7.2%; *P* <0.05, Figure 1). For COC maintained in medium when DRB was added 30 min after the initiation of culture, only 32.6 ± 7.2% (*P* <0.05) underwent GVBD compared to untreated controls. Progressively more oocytes underwent GVBD (Figure 1) when DRB was added at 60 (62.2 ± 7.2%), 90 (79.1 ± 7.2%), and 120 (90.8 ± 7.2%) min after the start of culture. The initial transcriptional events required for FSH-mediated GVBD, therefore, occurred between 0 and 30min of culture (Figure 1).

### Expression Patterns for Candidate mRNAs

**Figure 2:**
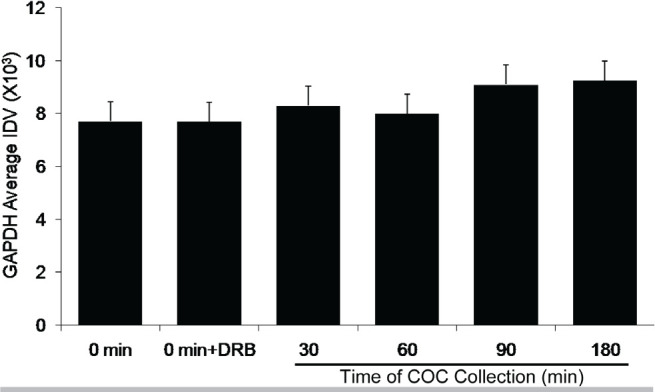
Expression of Gapdh mRNA, the housekeeping control, during the time course of COC collection. All treatments contained FSH (lsmeans ± SEM; n = 5 replicate experiments with n = 68 ± 3 COC per treatment per replicate).

**Figure 3:**
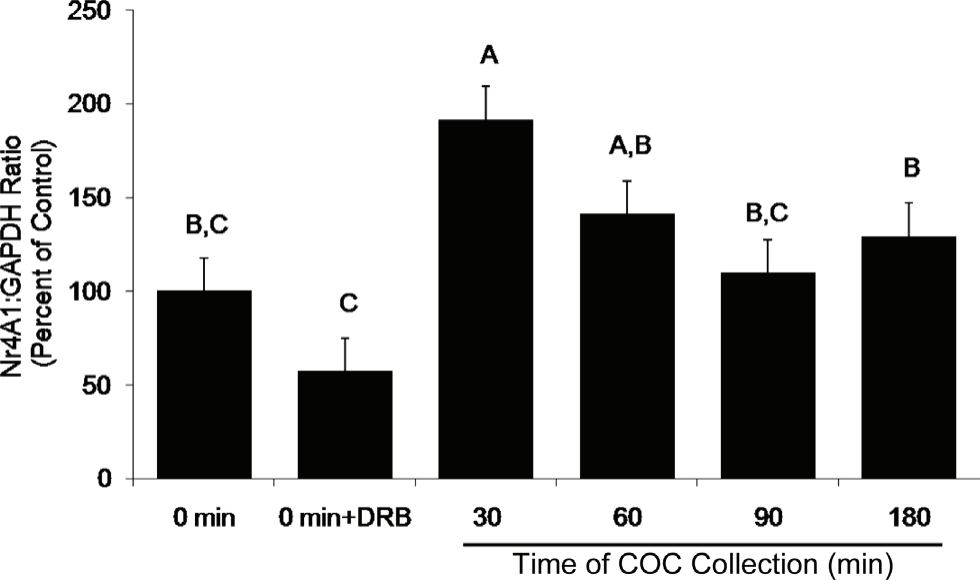
Pattern of expression for the candidate mRNA, Nr4A1, during the time course of COC collection. All treatments were cultured in the presence of FSH (lsmeans ± SEM; n = 5 replicate experiments with n = 68 ± 3 COC per treatment per replicate);^A,B,C^ *P* <0.05).

**Figure 4:**
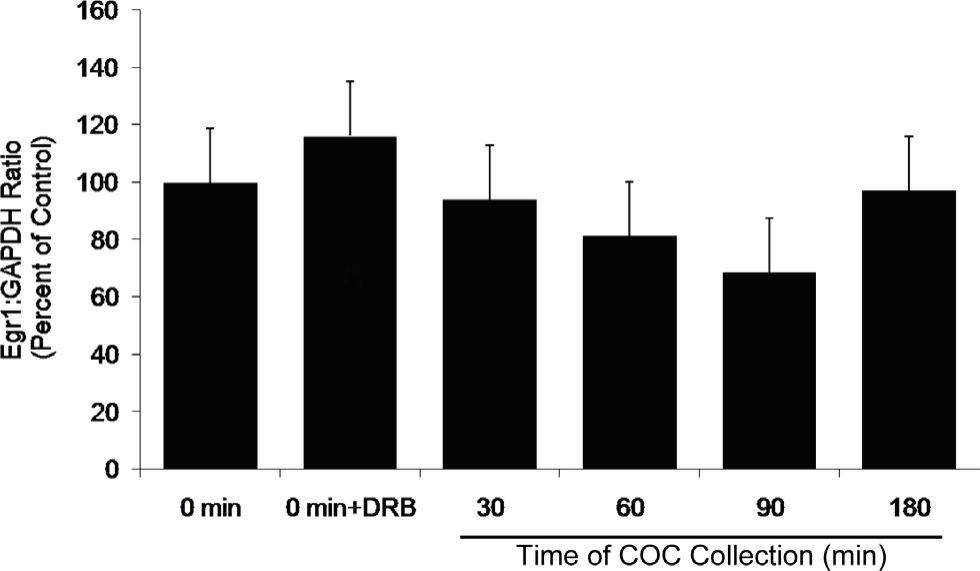
Pattern of expression for the candidate mRNA, Egr1, during the time course of COC collection. All treatments were cultured in the presence of FSH (lsmeans ± SEM; n = 5 replicate experiments with n = 68 ± 3 COC per treatment per replicate).

Subsets of COCs were collected at the start of culture and at 30, 60, 90, and 180min after culture initiation. COC were snap frozen for RNA extraction and subsequent mRNA analysis. A control group in which DRB was added from the start of culture and COCs collected at 180 min was also included. Subsets of COC from each culture group were cultured to 9 hr and assessed for the incidence GVBD to verify the effectiveness of DRB treatment to arrest GVBD. Levels of mRNA expression for the housekeeping mRNA, Gapdh, did not change during culture (Figure 2). This was expected since Gapdh mRNA is ubiquitous in the cytoplasm and its steady-state levels are not affected by DRB treatment under the conditions used. In contrast, levels of Nr4A1 mRNA increased significantly (*P* <0.05) at 30min after the start of culture (Figure 3), consistent with the time of transcription initiation required for GVBD (Figure 1). Levels of Nr4A1 mRNA slowly decreased in abundance throughout the remainder of culture (Figure 3). The increase in Nr4A1 mRNA expression seen at 30 min of culture did not occur if DRB was included at the initiation of COC culture (Figure 3). Levels of Egr1 mRNA did not change throughout the culture period and were not affected by the presence or absence of DRB during culture (Figure 4), implying that Egr1 mRNA was not transcriptionally regulated during this time course. Therefore, siRNA assessment of the potential role of Egr1 as a candidate mRNA involved in the transcriptional control of FSH-mediated bovine oocyte maturation was not pursued.

### siRNA Dose Response for Candidate Nr4A1

**Figure 5:**
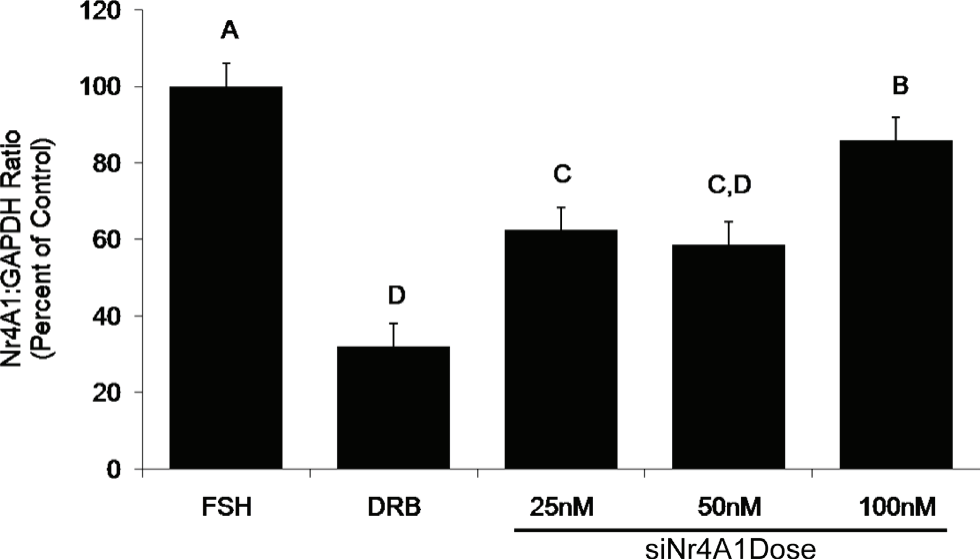
Effect of siRNA dose on expression of the candidate mRNA, Nr4A1. All treatment groups contain FSH (lsmeans ± SEM; n = 3 replicate experiments with n = 59 ± 4 COC per treatment per replicate;^A,B,C,D^ *P* <0.05).

Expression of Nr4A1 mRNA in COC cultured in the presence of FSH and either 25, 50, or 100nM siNr4A1 was assessed. Control treatments consisted of COC cultured in the presence of FSH alone or in the presence of FSH plus the transcriptional inhibitor, DRB. Expression of Nr4A1 mRNA was decreased compared to the FSH control in all treatment groups containing siNr4A1, with the greatest decreases occurring in the 25nM and 50nM siNr4A1 treatment groups (Figure 5).

### Effects of Nr4A1 siRNA Treatment on GVBD

Based on the results of the siNr4A1 dose response, the 50nM siNr4A1 dose was chosen for additional studies designed to examine the effectiveness of siNr4A1 for reducing the incidence of GVBD in cultured bovine COC.

**Figure 6:**
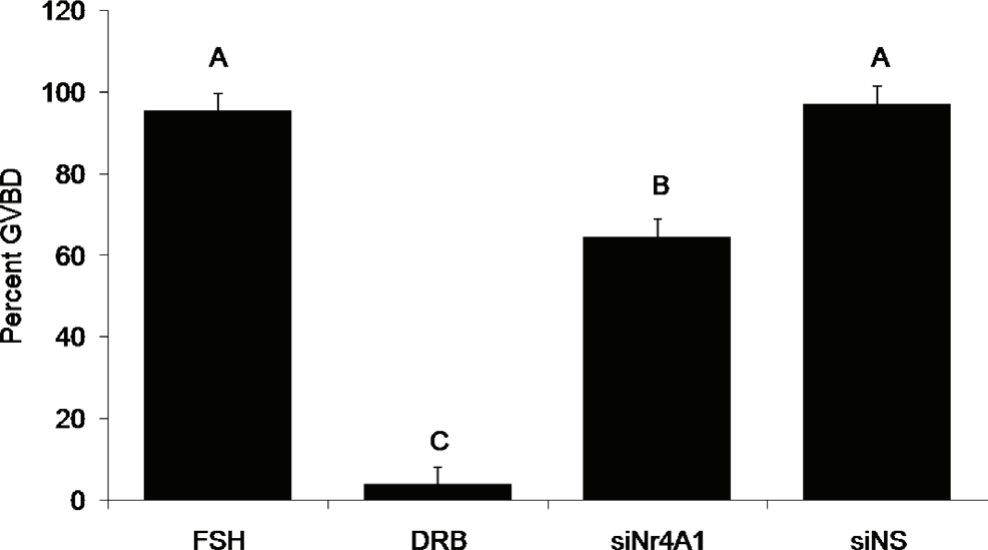
Effect of Nr4A1 siRNA on the percent of COC that underwent GVBD after 9 hrs of culture. All treatments contained FSH; siNr4A1 and siNS treatments used 50nM final concentration (lsmeans ± SEM; n = 4 replicate experiments with n = 13 ± 1 COC per treatment per replicate;^A,B,C^ *P* <0.05).

There was no significant difference in the proportion of COC that underwent GVBD at 9 hr of culture in the FSH control and non-specific small interfering RNA (siNS) treatment groups (Figure 6). As expected, significantly fewer COC cultured in the presence of the transcriptional inhibitor DRB underwent GVBD at 9 hr of culture (Figure 6). COC cultured with 50nM siNr4A1 showed a significant decrease in the percentage of oocytes undergoing GVBD after 9hr of culture (64.6 ± 4.2%) compared to COC cultured in FSH alone (95.4 ± 4.2%; *P* <0.05, Figure 6).

## DISCUSSION

Previous studies indicated that gene transcription required for gonadotropin-induced maturation of bovine COC in vitro occurred within 1 to 1.5 hr after the start of culture (Osborn & Moor, 1983; Kastrop *et al.*, 1991; Farin & Yang, 1994; Sirard *et al.*, 1998). In the present study, we have further refined the estimated time of transcription initiation required for FSH-induced bovine oocyte maturation in vitro. Based on our results, the critical period for transcription initiation in bovine COC begins as early as 30 min after the start of culture rather then at 60 to 90 min, as previously suggested (Farin & Yang, 1994; Sirard *et al.*, 1998). The candidate mRNAs selected for analysis in the current study were identified in earlier work by our group (Rodriguez *et al.*, 2006) in which SAGE libraries were designed to identify mRNA transcripts required for FSH-induced GVBD in cultured murine COC. Two of these candidate mRNAs, Nr4A1 and Egr1, both demonstrated decreased expression when murine COC were cultured in the presence of FSH and the inhibitor DRB compared to culture with FSH alone (Rodriguez *et al.*, 2006). Nr4A1 is a transcription factor classed as an immediate-early response gene whose expression is rapidly induced by a variety of physiological stimuli (Martin and Tremblay, 2010; Eells *et al.*, 2000) and has been identified in reproductive tissues including granulosa cells (Park *et al.*, 2003; He *et al.*, 2013) and Leydig cells (Martin *et al.*, 2009). Expression of Nr4A1 can be induced in response to LH in rat granulosa cells (Park *et al.*, 2003) and mouse Leydig cells (Song *et al.*, 2001), to FSH in human granulosa-lutein cells (He *et al.*, 2013), rat ovarian tissue (Schmidt *et al.*, 2006) and rat Sertoli cells (McLean *et al.*, 2002), and by cAMP (Kovalovsky *et al.*, 2002; Maira *et al.*, 2003; Pirih *et al.*, 2005; Martin *et al.*, 2009). Nr4A1 upregulation has also been associated with the induction of steroidogenic pathways (Mangelsdorf *et al.*, 1995; Martin & Tremblay 2005) and inhibin secretion (He *et al.*, 2013).

Egr1 has been implicated as a vital mRNA in oocytes, having been identified in bovine granulosa cells and found to have a profound effect on oocyte developmental competence (Robert *et al.*, 2001). Egr1 is also thought to be induced by FSH and plays an active role in follicular growth and ovulation (Fritz *et al.*, 2002), as well as in oocyte maturation by regulating closure of gap junctions (Russell *et al.*, 2003).

Analysis of expression profiles for Nr4A1 in bovine COC demonstrated that Nr4A1 mRNA levels increased approximately 30 min after the start of culture.

The apparent correlation between Nr4A1 levels increasing at 30 min after the start of culture and the start of transcription initiation required for FSH-induced in vitro maturation supports the suggestion that Nr4A1 is not only present in bovine COC, but also may play a role in the resumption of meiosis. In contrast to Nr4A1 mRNA, levels of bovine Egr1 mRNA did not increase during this critical time period. Thus, Egr1 does not appear to play a vital role in initiating GVBD in bovine COC. Additionally, it should be noted that Egr1 mRNA expression was not sensitive to the transcriptional inhibitor DRB. This finding suggests that, in cattle, Egr1 is not a nascent mRNA produced during the initial transcriptional events required for FSH-mediated GVBD.

Culture of bovine COC with siRNA targeting Nr4A1 mRNA resulted in a decreased proportion of COC undergoing GVBD after 9 hrs of culture as compared to COC cultured in the presence of non-specific siRNA. These findings suggest that Nr4A1 may play a role in FSH-mediated resumption of meiosis and subsequent GVBD in cultured bovine COC.

There was an incomplete inhibition of GVBD at 9hr of culture in the presence of the siNr4A1 compared to DRB. It is likely that inhibition of a single mRNA species by the siNr4A1 treatment may not be sufficient to fully prevent GVBD. In contrast, DRB treatment was completely effective in blocking GVBD; however, DRB blocks transcription of all nascent mRNAs, not just one specific mRNA. Thus, our observations suggest that while siNr4A1 treatment is partially effective in arresting GVBD, Nr4A1 may not be the only nascent mRNA transcript involved.

An alternative explanation for the partial effectiveness of the siNr4A1 treatment in arresting GVBD may be associated with uneven transfection of the siRNA into the bovine COC. Some COC have thicker, denser layers of cumulus cells and it is likely that the siNr4A1 penetrated the cumulus cell investments unevenly in those COC. Thus, it is possible that COC with more densely packed layers of cumulus cells may not have been transfected with the siNr4A1 to the same degree as COC with fewer cumulus layers, potentially contributing to incomplete inhibition of GVBD observed with the siNr4A1 treatment. Because the oocyte and its surrounding cumulus cells form a single physiological unit that must be maintained as an intact structure for gonadotropin-induced oocyte maturation to occur, we chose to use COC, rather than denuded oocytes for these experiments. Messenger RNAs regulating the initiation of GVBD would be expected to be products of the cumulus cells, rather than products of the oocyte (Rodriguez & Farin, 2004). In this regard, it may be useful in future studies to use microinjected, denuded oocytes as an additional control group to examine the effectiveness of siNr4A1 to inhibit GVBD. This would permit further confirmation that the site of the transcriptional activity involved in mediating FSH-induced GVBD is the cumulus cell investment.

In summary, gene transcription required for GVBD in cultured bovine COC occurs as early as 30min after culture initiation and expression of Nr4A1 mRNA increases coincident with the time during which transcription is required for FSH-induced GVBD in vitro. Furthermore, while Egr1 mRNA may be present in bovine COC, Egr1 mRNA levels did not change throughout culture nor were they affected by treatment with a transcriptional inhibitor.

Expression of Nr4A1 mRNA was decreased in COC cultured in the presence of siNr4A1 and culture of COC with siNr4A1 decreased the percentage of oocytes undergoing GVBD after 9hr of culture. In conclusion, Nr4A1 appears to play a role in regulating FSH-induced GVBD in cultured bovine COC.
